# Tackling a Major Deficiency of Diversity in Alzheimer’s Disease Therapeutic Trials: An CTAD Task Force Report

**DOI:** 10.14283/jpad.2022.50

**Published:** 2022-05-13

**Authors:** Rema Raman, P. Aisen, M. C. Carillo, M. Detke, J. D. Grill, O. C. Okonkwo, M. Rivera-Mindt, M. Sabbagh, B. Vellas, M. Weiner, R. Sperling, CTAD Task Force

**Affiliations:** 1 Alzheimer’s Therapeutic Research Institute, Keck School of Medicine, University of Southern California, San Diego, CA 92121 USA 9860 Mesa Rim Road,; 2 Alzheimer’s Association, Chicago, IL USA; 3 Cortexyme, South San Francisco, CA USA; 4 Institute for Memory Impairments and Neurological Disorders, University of California Irvine, Irvine, CA USA; 5 Wisconsin Alzheimer’s Disease Research Center and The Department of Medicine, University of Wisconsin School of Medicine And Public Health, Madison, WI USA; 6 Neurology, Icahn School of Medicine at Mount Sinai, New York, NY USA; 7 Psychology & Latin American Latino Studies Institute, Fordham University, Bronx, NY USA; 8 Barrow Neurological Institute, Phoenix, AZ USA; 9 Gerontopole of Toulouse, Institute of Ageing, Toulouse University Hospital (CHU Toulouse), Toulouse, France; 10 University of California, San Francisco, CA USA; 11 Department of Neurology, Massachusetts General Hospital and Harvard Medical School, Boston, MA USA

**Keywords:** Alzheimer’s disease, clinical trials, participant diversity, generalizability

## Abstract

As the last opportunity to assess treatment effect modification in a controlled setting prior to formal approval, clinical trials are a critical tool for understanding the safety and efficacy of new treatments in diverse populations. Recruitment of diverse participants in Alzheimer’s Disease (AD) clinical trials are therefore essential to increase the generalizability of study results, with diversity broadly described to be representative and inclusive. This representation of study participants is equally critical in longitudinal cohort (observational) studies, which will be key to understanding disease disparities and are often used to design adequately powered AD clinical trials. New and innovative recruitment initiatives and enhanced infrastructure facilitate increased participant diversity in AD clinical studies.

## Introduction

**T**he COVID-19 pandemic has highlighted and exacerbated health inequities globally. Health and healthcare disparities are sustained when clinical research, including observational studies and clinical trials, fail to recruit samples that are representative and generalizable. In the United States, disparities based on social constructs of race and ethnicity, as well as age, sex, educational attainment, and socioeconomic status are striking (1–3). In 2020, in industry-sponsored trials that supported Food and Drug Administration (FDA) approval of drugs and biologics, only 8% of participants were Black or African American, 6% Asian, and 11% Hispanic or Latino(a) (4). Diversity in terms of racial and ethnic representation in clinical trials participation is similarly poor in other regions of the world as well (5).

The situation is worse in Alzheimer’s disease (AD) research. The risk and burden of AD are greater among African Americans/Blacks and Hispanics/Latinos, compared to non-Hispanic Whites; yet these groups and other racial and ethnic groups are underrepresented both in AD observational studies (6) and randomized clinical trials (7). In the aducanumab trials, the first new drug approved by the FDA in over 20 years of research and development, only 0.6% of participants were of African American/Black race and 1.5% were of Hispanic/Latino(a) ethnicity (8).

Making studies more inclusive and representative is imperative. Recognizing this as a decisive moment in AD research, the EU/US/Clinical Trials in Alzheimer’s Disease (CTAD) Task Force, an international collaboration of AD investigators across industry and academia, met November 2021 to review current data, describe new and unique diversity initiatives being implemented in the United States (US) to address this problem, and combine efforts that will immediately support the inclusion of representative populations in US AD cohort studies and clinical trials.

## National Strategy

In 2019, the US National Institute on Aging (NIA), in collaboration with the Alzheimer’s Association, established the National Strategy for Recruitment and Participation in Alzheimer’s and Related Dementias (ADRD) Clinical Research (9). The goal was to engage broad segments of the public in the AD and AD Related Dementias (ADRD) research enterprise, with a particular focus on underrepresented communities, to enroll and retain individuals in clinical research studies successfully and more quickly. This strategy had four broad areas of focus: (a) Increase awareness and engagement at a broad, national level, (b) Build and improve capacity and infrastructure at the study site level, (c) Engage local communities and support participants, and (d) Develop an applied science of recruitment. The NIH UNITE initiative was established to identify and address structural racism within the NIH-supported research and the greater scientific community. Multiple tools were developed for the global AD research community, including an ADRD recruitment planning guide, an NIA repository of Alzheimer’s & Dementia Outreach, Recruitment & Engagement Resources (ADORE), and a suite of tools to build or enhance outreach effort (OUTREACH PRO). More recently, an effort to accelerate access to NIA-funded clinical research enrollment data led to the establishment of the Clinical Research Operations and Management System (CROMS) to provide real-time tracking, reporting, and management of clinical research enrollment data.

Several training programs have been established to diversify the ADRD clinical research workforce (10). These include predoctoral, postdoctoral and junior faculty Fellowship awards as well as a training program to diversify the next generation of ADRD clinical trialists (11).

## Observational Studies

Over the past several decades, multiple longitudinal observational studies have been conducted to better characterize the progression of the disease across the AD spectrum, from autosomal-dominant to early-onset to sporadic AD. The primary aim of several of these observational studies is to improve clinical trial design, develop better trial endpoints, and to discover and validate biomarkers for AD clinical research. Participant populations in these studies are consistently homogeneous, namely, White race, non-Hispanic ethnicity and having over 12 years of formal education. Lack of representation in these studies is highly problematic as it could lead to inaccurate and biased disease progression models, which then lead to sub-optimal clinical trial study designs and outcomes. Discussion at the Task Force meeting centered around some early successes in changing this demographic through innovative recruitment initiatives.

### New IDEAS

The New IDEAS study, built upon the IDEAS study platform (12), is a national, open-label study to determine the utility of a brain amyloid PET scans in more accurate diagnosis and better treatment decisions. Seeking to understand the differences by race/ethnicity in positive amyloid PET scan proportions, this study will enroll a diverse cohort of 7000 Medicare beneficiaries, with at least 2000 Black/African American and at least 2000 Hispanic/Latino(a) participants with early-onset or late-onset dementia and typical and atypical presentations of AD. The study will use a comprehensive recruitment strategy using both general and study level barriers including lack of study awareness, study materials for families involved in decision making and co-pays (13, 14).

### ADNI

The Alzheimer’s Disease Neuroimaging Initiative (ADNI) was established as an observational study to instruct clinical trials and develop standardized imaging techniques and biomarker procedures in normal participants, participants with MCI, and participants with mild AD. The current version of ADNI, ADNI-3 (15), continues follow existing and new participants with the goal of informing AD clinical trial design.

General recruitment strategies have included online advertisements, local radio and newspaper coverage, and emails and referrals from patient registries (16), like the Brain Health Registry (17). Additionally, TV campaigns with community influencers such as B. Smith (a Black former model, restauranter, and TV host diagnosed with AD) and development of a Latino(a) focused participant registry aimed to improve study diversity. With these efforts, the current composition of the ADNI sample is 89% White, with 5% Black/African American, 8% Hispanic/Latino(a), and 3% Asian. Additionally, only 15% of the participants have education levels of 12 years or lower.

To ensure that the findings from ADNI3 would be generalizable, improved diversity will be key. To that end, a diversity task force (DVTF) was established with the aim of increasing the recruitment and engagement of participants from underrepresented populations, with a focus on participants of Black/African American race and Hispanic/Latino(a) ethnicity. Key elements to this task force approach included (a) a community-engaged research approach, (b) engagement and support of 13 DVTF clinical sites, (c) changes to protocol design including optional lumbar punctures and remote assessments and (d) applying a health equity lens to the existing ADNI data. Initial results were promising. The rate of URP enrollment into ADNI increased from 1.1 participants/month before DVTF efforts to 4 participants/month after DVTF efforts. Of the 43 enrollments since February 2021, 10 (23%) self-identified to URPs.

Recommendations from the ADNI DVTF initiative include:

- Have study investigators from URPs in leadership.
- Establish community advisory board(s), as well as a scientific advisory board
- Use marketing firms with community ownership and experience
- Important to have local site participation
- Incorporate community-engaged digital marketing to centralized recruitment initiatives

## Clinical Trials

Clinical trials are the last step before potential approval in understanding how new treatments might impact diverse populations. Yet, most AD trial participants from the United States are of White race, non-Hispanic ethnicity, have over 12 years of formal education, are married, and enroll with a spousal study partner (7, 18, 19). Recruitment approaches being utilized, and infrastructure being established to increase the enrollment of diverse participants into trials were discussed at the Task Force meeting.

### ACTC

The Alzheimer’s Clinical Trials Consortium (ACTC) is a U.S-based NIH-funded national consortium established to accelerate the development of effective interventions for AD and related disorders (ADRD). Through a formal recruitment unit, the goals of the ACTC include the development and implementation of cutting-edge strategies and tactics to accelerate and make more efficient participant accrual and randomization, increase trial representativeness through enhanced diversity, and maximize participant retention across the spectrum of AD clinical trials. The approach is site and participant-focused, culturally sensitive, data-driven, and evidence-based and includes a four-part strategy:

- Establishment of infrastructure: In addition to the establishment of the Recruitment Unit, ACTC member sites are provided infrastructure funding that increases expectations toward site performance in trial start-up and participant recruitment. Additional aspects of the ACTC infrastructure include a diverse centralized recruitment team, participation of the Unit leadership in the earliest stages of trial design, beginning with proposal development and carrying through protocol design, establishment of inclusion/exclusion criteria, site selection with a focus on diverse recruitment (including site capacity to enroll monolingual Spanish speakers), centralized support for recruitment and retention activities for all trial sites, and the development of a recruitment and retention plan for each specific trial.
- Comprehensive outreach and engagement model: ACTC sites use multiple recruitment strategies, including well established local efforts but also key support from the central team. These include the development of a study website that can serve as a key landing spot for recruitment activities and inform the public about a specific trial. The Unit develops a catalog of recruitment materials in multiple languages such as study brochures, community presentations, educational materials, letters to the editor for use by site teams in their local markets. Central efforts include attempts to increase national, regional, and local public awareness through earned media (with professional partners when budgets permit). These include placement of stories about a trial on television, radio, and newspaper outlets, as well as novel efforts to reach participants through things like grocery store advertisements. The Unit also utilizes a Hub-and-Spoke model to support an innovative and deep collaboration with non-academic experts in health disparities and community engagement. The “Hub” features leadership with extensive experience in ADRD clinical trials as well as community-based participant advocacy. These Hub investigators, work closely with select “Spoke” site recruitment teams to provide sustained and intensive guidance, with the goal of building and enhancing community partnerships, avoiding common pitfalls, and ultimately improving recruitment of underrepresented communities into trials at their sites.
- Recruitment Science: The Unit takes a data-driven, evidence-based approach to evaluating the success of recruitment strategies and guiding future approaches. Recent work has shown that there are racial and ethnic differences in the frequency of screen failure in preclinical AD trials and noted differential rates of exclusion based on cognitive as well as biomarker requirements (19). Disparities such as these likely result from genetic (20) as well as social/environmental causes such as co-morbidity rates and challenges resulting from cognitive testing in a second language. Addressing risk for differential exclusion requires a study-specific approach (21) but also a consistent focus on and effort toward inclusivity.
- Establishment of a pre-screening database and minimal dataset to be used in all ACTC trials. Participant recruitment equates to a large funnel, with top of that funnel representing all potentially eligible participants. The funnel narrows based on trial awareness, willingness to participate, and eligibility. Traditionally, recruitment data focus on screening rates, but understanding the impact of recruitment efforts on awareness and initial interest in trials may facilitate recruitment intervention designs and resource expenditures. The ACTC pre-screening database captures site activities in the forms of participant calls, referrals from registries and other sources, and other forms of initial contact that may move earliest in response to central recruitment campaigns or reveal potential differential exclusion of specific groups or communities, even before consenting, enrollment, and screening. Once participants are screened for trials, a common minimal data set can be used to facilitate meta-analyses across network trials, including assessments of recruitment, retention, and trial inclusivity. Ensuring careful and consistent assessment of race, ethnicity, and potential social determinants of health may reveal important trends or opportunities in recruitment and retention.

### U. S. POINTER

The U.S. Study to Protect Brain Health through Lifestyle Intervention to Reduce Risk (U.S. POINTER) is a Phase 3, two-year clinical trial to evaluate whether lifestyle interventions that simultaneously target multiple risk factors protect cognitive function in older adults at increased risk for cognitive decline conducted across 5 sites within the United States. U.S. POINTER employs multiple approaches to meet the diverse recruitment study goals, including, grassroots recruitment structure, staff education and leadership support, working with Institutional review boards, developing toolkits for network-based outreach, a 3-tier approach for constant engagement, and through the activation of community advocates.

## Conclusions/Next Steps

While challenges continue to exist in the enrollment of a diverse sample in AD clinical studies, addressing this issue for the field is now a priority across academia, industry, non-profit and government agencies. To meet the challenges in recruiting a representative sample in US ADRD clinical trials, intentional and sustained efforts are needed. Future ADRD clinical trials must include clear, measurable, and attainable goals for diverse enrollment with attention to these issues from the point of study planning through design and conduct, with adequate resources for site and community engagement. Evidence-based recruitment science should be incorporated to better understand the reasons and sources of participant bias and whether it differs across sub-populations. There is much progress in the field with the recent guidance by the Food and Drug Administration that emphasizes the need for diversity of clinical trials participants (21) and the Equity in Neuroscience and Alzheimer’s Clinical Trials Act (ENACT) being considered by the United States Congress to increase the participation of underrepresented populations in Alzheimer’s and other dementia clinical trials. Similar efforts are ongoing in the United Kingdom, across Europe, Latin America and in the Asia-Pacific region. Continued global collaborations and open sharing of data, knowledge, and experiences across all regions of the world will be needed to establish inclusive and representative study samples in national and global AD clinical trials.
